# Surface-Relief Gratings in Halogen-Bonded Polymer–Azobenzene Complexes: A Concentration-Dependence Study

**DOI:** 10.3390/molecules22111844

**Published:** 2017-10-28

**Authors:** Jelle E. Stumpel, Marco Saccone, Valentina Dichiarante, Ossi Lehtonen, Matti Virkki, Pierangelo Metrangolo, Arri Priimagi

**Affiliations:** 1 Laboratory of Chemistry and Bioengineering, Tampere University of Technology, P.O. Box 541, FI-33101 Tampere, Finland; jelle.stumpel@tut.fi (J.E.S.); marco.saccone84@gmail.com (M.S.); matti.virkki@tut.fi (M.V.); 2 Department of Applied Physics, Aalto University, P.O. Box 15100, FI-00076 Espoo, Finland; ossi.lehtonen@aalto.fi (O.L.); pierangelo.metrangolo@polimi.it (P.M.); 3 Laboratory of Supramolecular and Bio-Nanomaterials (SupraBioNanoLab), Department of Chemistry, Materials and Chemical Engineering “Giulio Natta”, Politecnico di Milano, Via L. Mancinelli 7, I-20131 Milano, Italy; valentina.dichiarante@polimi.it

**Keywords:** surface-relief grating, azobenzene, halogen bonding, supramolecular, photoresponsive

## Abstract

In recent years, supramolecular complexes comprising a poly(4-vinylpyridine) backbone and azobenzene-based halogen bond donors have emerged as a promising class of materials for the inscription of light-induced surface-relief gratings (SRGs). The studies up to date have focused on building supramolecular hierarchies, i.e., optimizing the polymer–azobenzene noncovalent interaction for efficient surface patterning. They have been conducted using systems with relatively low azobenzene content, and little is known about the concentration dependence of SRG formation in halogen-bonded polymer–azobenzene complexes. Herein, we bridge this gap, and study the concentration dependence of SRG formation using two halogen-bond-donating azobenzene derivatives, one functionalized with a tetrafluoroiodophenyl and the other with an iodoethynylphenyl group. Both have been previously identified as efficient molecules in driving the SRG formation. We cover a broad concentration range, starting from 10 mol % azobenzene content and going all the way up to equimolar degree of complexation. The complexes are studied as spin-coated thin films, and analyzed by optical microscopy, atomic force microscopy, and optical diffraction arising during the SRG formation. We obtained diffraction efficiencies as high as 35%, and modulation depths close to 400 nm, which are significantly higher than the values previously reported for halogen-bonded polymer–azobenzene complexes.

## 1. Introduction

Photoinduced surface patterning is considered to be among the most important macroscopic effects triggered by the photoisomerization reaction in azobenzene-containing polymers [1]. It dates back to 1995 when it was observed that, upon irradiating an azopolymer thin film with an optical interference pattern, the polymer starts to migrate and forms a replica of the incident irradiation pattern on the polymer surface in the form of a surface-relief grating (SRG) [2,3]. Once inscribed, these diffraction gratings are temporally stable at room temperature, yet can be erased either thermally or with light and subsequently reconfigured [4,5]. Due to the versatility and ease of fabrication, the SRGs show potential for a plethora of applications in photonics and nanotechnology [6,7].

Even if the theoretical foundations for SRG formation are being continuously elaborated [8,9,10], the mechanism and structure-performance relations behind these light-induced macroscopic motions are not yet perfectly understood. However, it is well established that, in polymeric systems, efficient SRG formation requires specific bonding interactions between the photoactive azobenzene units and the polymer matrix [11,12,13]. This can take place either through covalent bonding, as in the case of, e.g., poly(disperse red 1 Acrylate) [2], or via strong and/or specific non-covalent interactions such as ionic bonding [14,15,16], hydrogen bonding [13,17,18], or—as in the case studied in the present paper—halogen bonding [19,20].

It is also known that the concentration of the azobenzene molecules bound to the polymer matrix greatly affects the overall efficiency of the SRG inscription [21]. The concentration-dependence studies are greatly facilitated by supramolecular functionalization strategies, and several studies have been conducted using hydrogen-bonded polymer–azobenzene complexes, however, with somewhat inconclusive results. Some studies report linear increase in surface patterning efficiency as a function of concentration [17,18], whereas others report saturation of SRG formation above some threshold concentration [22,23]. Additionally, the minimum amount of azobenzene needed for photoinscription of SRGs has been investigated using hydrogen-bonded polymer–azobenzene complexes [24]. To the best of our knowledge, the concentration dependencies in other types of supramolecular systems, such as those that are halogen-bonded, have not been elaborated.

Halogen bonding (XB) appears as a particularly versatile noncovalent interaction in designing high-performance, azobenzene-based photoresponsive materials [25]. It is defined as a noncovalent interaction between an electrophilic region on a halogen atom (σ-hole [26]) and a nucleophilic region on another, or the same, molecule [27]. The main attractive feature of XB is its high directionality as compared to hydrogen bonding [28], allowing it to co-exist or compete with the latter even if its interaction strength with the bond acceptors would be weaker [29]. Beautiful examples on the prevalence or the coexistence of halogen bonds over hydrogen bonds in, e.g., crystal engineering [30] and anion recognition in solution [31] have appeared in the literature.

**Figure 1:**
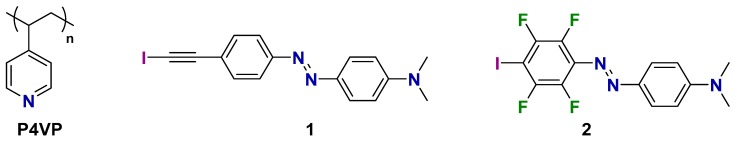
Chemical structures of the halogen-bond-accepting polymer (P4VP) and the halogen-bond-donating azobenzene derivatives (Molecules **1** and **2**) used in this work.

We have studied extensively the potential use of halogen-bonded supramolecular polymers for photoinduced surface patterning [19,20]. In these studies, we have utilized a molecular library of azobenzenes substituted with dimethylamino groups to promote SRG formation [32,33], and with halogen- and hydrogen-bond donor groups in order to build supramolecular hierarchies and better understand the optimal choice of polymer–azobenzene noncovalent interactions for efficient light-induced surface patterning. Due to a higher degree of photo-induced alignment of the chromophores and polymer chains [34], halogen-bonded polymer–azobenzene complexes outperform their hydrogen-bonded counterparts at least when using moderate azobenzene concentrations, and two molecules, **1** and **2** in Figure 1, appeared as particularly promising for driving SRG formation. Herein, we extend our studies towards the effect of concentration on SRG formation efficiency in halogen-bonded supramolecular polymers, an important step towards optimizing the material composition for SRG-forming supramolecular systems, and in gaining further knowledge on the structure–property relations behind this intriguing photomechanical phenomenon.

## 2. Results and Discussion

We compare SRG formation in supramolecular polymer–azobenzene complexes containing the halogen-bond-donating azobenzenes **1** and **2** (Figure 1), incorporated into halogen-bond-accepting polymer poly(4-vinylpyridine) (P4VP). This polymer is widely used to devise self-assembled supramolecular polymeric materials [35,36,37]. It functions as an acceptor for both halogen bonds and hydrogen bonds, and has been our workhorse polymer for photoinduced surface patterning studies [5,19,20,22,23,24,33]. The iodoethynylphenyl-based and tetrafluoroiodophenyl-based azobenzene derivatives (**1** and **2**, respectively) were chosen based on our previous studies [19,20]. At low azobenzene concentrations, both **1** and **2** are efficient in driving SRG formation in P4VP. We hypothesize, however, that their performance as a function of concentration might be very distinct. This is because we expect **2** to be more prone to phase separation and aggregation at high concentrations due to quadrupolar interactions, driven by the packing of fluorinated and non-fluorinated rings [38,39,40]. Clarifying this issue is the very reason for undertaking this study.

**Figure 2:**
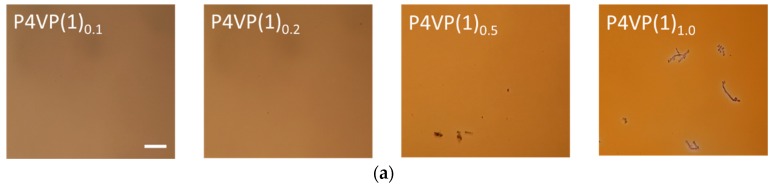

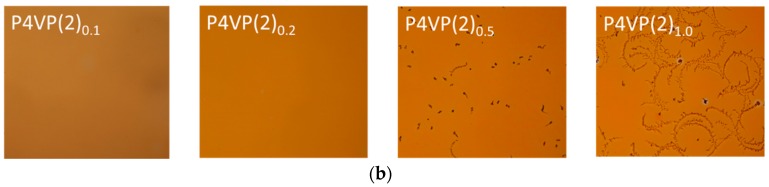
Optical microscope images (magnification 10×) of thin films of the halogen-bonded (**a**) P4VP(**1**)***_x_*** and (**b**) P4VP(**2**)*_x_* complexes, with complexation degrees *x* equaling to 0.1, 0.2, 0.5, and 1.0. Phase separation was clearly observed at high complexation degrees, especially for the **2**-containing samples. The scale bar represents 100 μm.

To test our hypothesis, we prepared mixtures with complexation degrees of 0.1, 0.2, 0.5, and 1.0 (referring to the number of azobenzenes with respect to the polymer repeat units) for further investigations. From here on, the samples will be denoted as P4VP(**1**)***_x_*** and P4VP(**2**)*_x_*, where *x* stands for the complexation degree. The complexes were studied as thin films prepared by spin coating on clean microscope slides (see Table 1 for film thicknesses). The film quality was monitored with an optical microscope, as shown in Figure 2. In line with our previous studies, the low-concentration samples (*x* ≤ 0.2) were clear and amorphous, showing no sign of crystallization or phase separation. At higher concentrations, the differences between **1** and **2** became evident, and phase separation was clear for P4VP(**2**)_0.5_ and P4VP(**2**)_1.0_, the latter of which was easily observable with the naked eye directly after spin coating. This behavior was absent in P4VP(**1**)_0.5_ and much less pronounced in P4VP(**1**)_1.0_, indicating that indeed the phenyl-perfluorophenyl structure of **2** promotes phase separation between the polymer and the azobenzene dopants.

**Figure 3:**
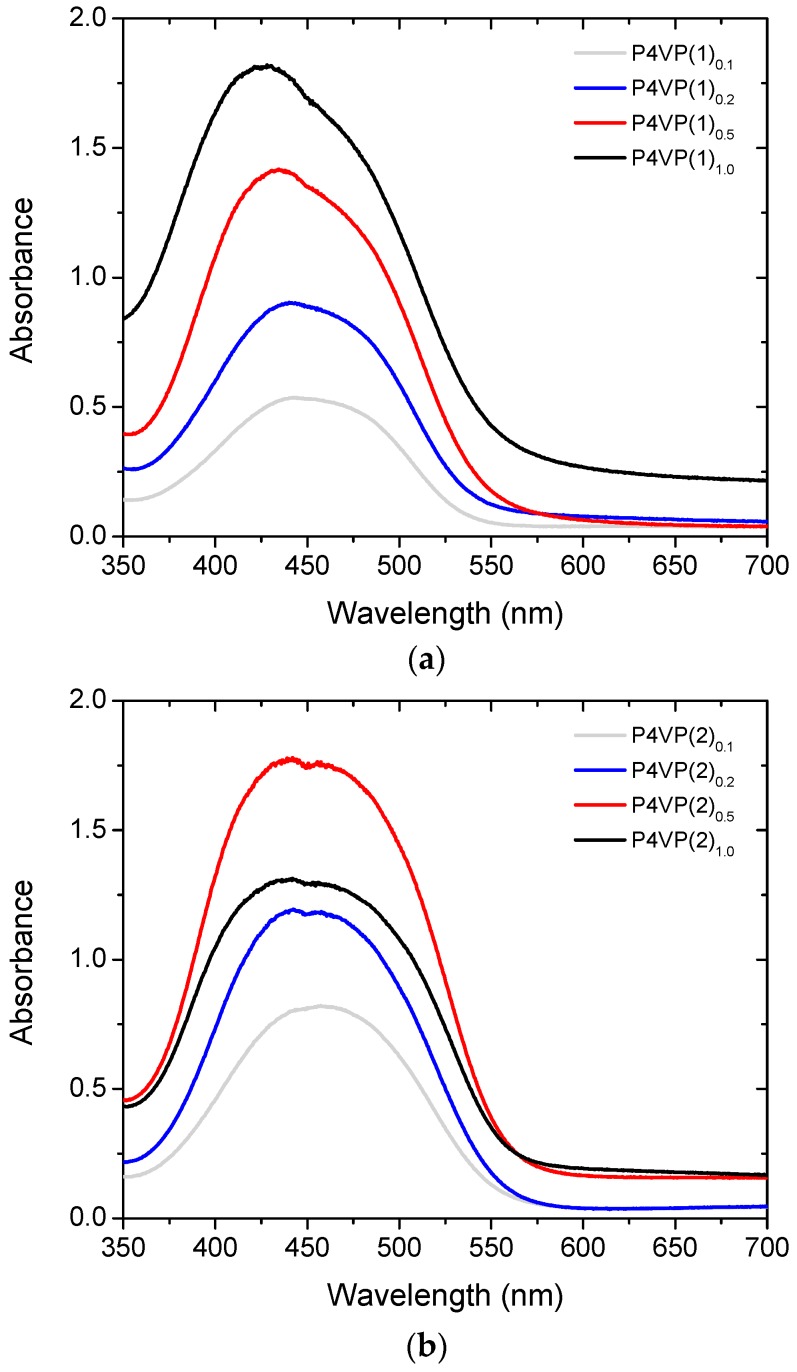
Absorption spectra of thin films of halogen-bonded (**a**) P4VP(**1**)***_x_*** and (**b**) P4VP(**2**)*_x_* complexes with complexation degree *x* = 0.1, 0.2, 0.5, and 1.0. The data has been normalized to match with a thickness of 400 nm.

We next measured the absorption spectra of the samples (from the same thin films whose optical microscope images are shown in Figure 2), as presented in Figure 3. At the complexation degree *x* = 0.1, the spectra are very similar to those measured in dilute solution, indicating that the molecules at this concentration range are essentially isolated from one another. The absorption maximum of **2** (457 nm) is somewhat red-shifted as compared to **1** (443 nm), which can be attributed to the presence of the electron-accepting fluorine atoms that increase the push–pull character of the compound. Upon increasing the complexation degree, the absorbance of the samples naturally increases, but in addition, for **1** a clear blue-shift (λ_max_ = 443→427 nm) is observed. This is a consequence of intermolecular interactions (side-by-side packing) between the azobenzene units. However, the blue-shift does not necessarily indicate macroscopic phase separation and occurs even when the azobenzene units are attached to polymer chains, either covalently [41,42] or non-covalently [43]. In P4VP(**2**)_0.1→1.0_, the blue-shift is less noticeable, but the absorption band is significantly broadened at high complexation degrees, also an indication of intermolecular interactions between the azobenzenes. In addition to spectral changes at high degrees of complexation, optical scattering—seen as an increase in the baseline of the spectra (600–700 nm)—is significant for samples P4VP(**1**)_1.0_, P4VP(**2**)_0.5_, and P4VP(**2**)_1.0_. This is well in line with the optical micrographs depicted in Figure 2, indicating that the scattering domains arise from the phase-separated azobenzene chromophores.

Based on the optical micrographs and the UV-Vis spectra, we could identify clear differences between the two sample series, suggesting that our initial hypothesis on the role of fluorine on phase separation is correct. Azobenzene aggregation is known to affect photoinduced anisotropy that can be generated upon polarized light irradiation in a complicated, intensity-dependent manner [44]. However, there is no well-established connection between the aggregation and the SRG formation efficiency, the subject of our next experiments. An interference pattern was created using a spatially filtered, circularly polarized beam with 300 mW·cm^−2^ intensity from a 488 nm laser in a Lloyd mirror configuration, with a spatial period of 1 μm. We chose this period as it is optimal in writing high-modulation-depth SRGs in azobenzene polymer films [45]. The time evolution of SRG formation was monitored by detecting the transmitted first-order diffracted beam from a normally incident 633 nm He–Ne laser.

**Figure 4:**
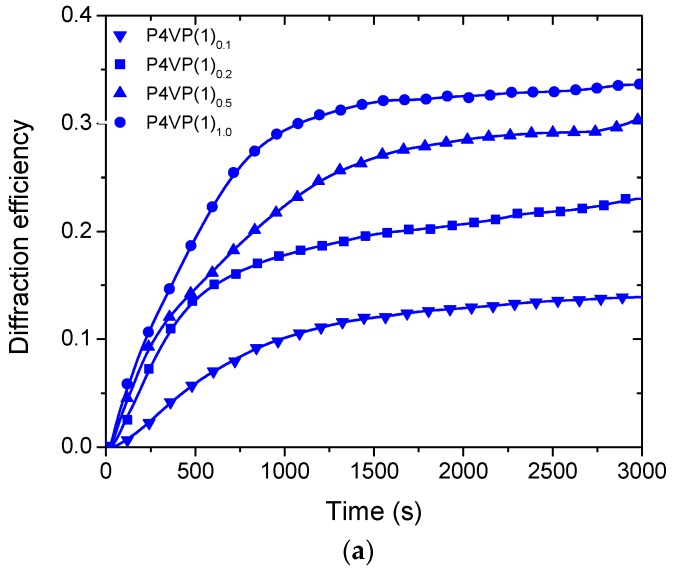

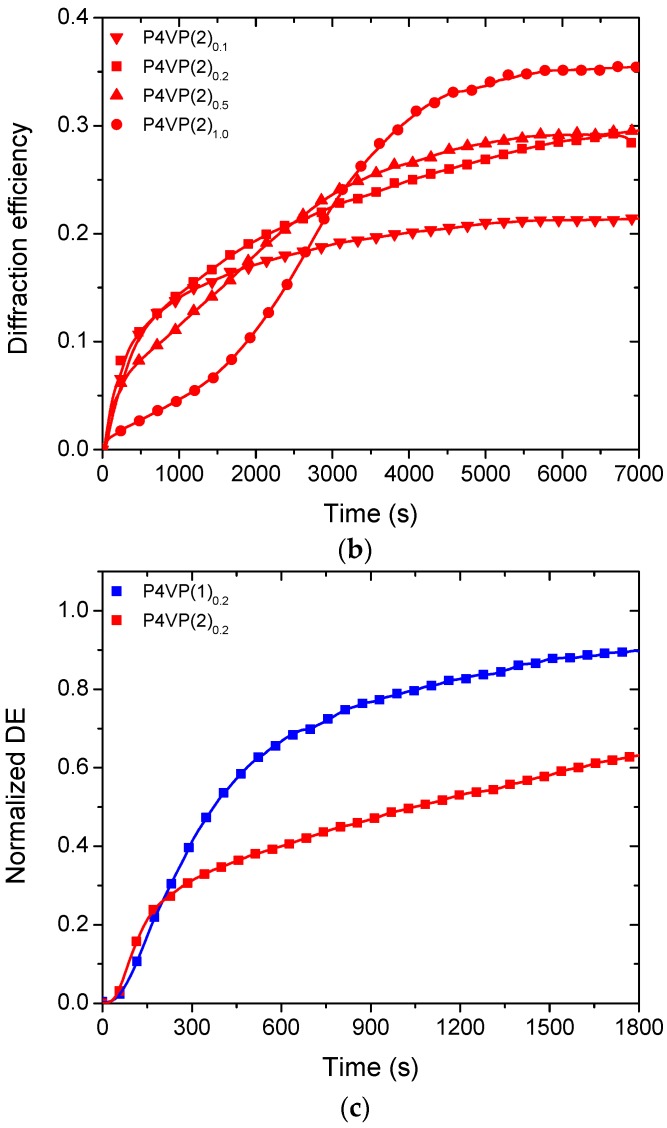
Development of first order diffraction efficiencies during SRG formation with a writing beam irradiation intensity of 300 mW·cm^−2^ (488 nm) for (**a**) P4VP(**1**)*_x_* and (**b**) P4VP(**2**)*_x_*; and (**c**) the normalized diffraction efficiencies (0–1800 s) during SRG formation for the samples with nominal complexation degree *x* = 0.2.

The first-order diffraction efficiencies for P4VP(**1**)*_x_* and P4VP(**2**)*_x_* during SRG inscription are presented in Figure 4a,b, respectively. Several notable differences between the two cases can be identified. First of all, irrespective of the azobenzene concentration, the diffraction efficiency saturates faster in case of using **1** as the photoactive unit (Figure 4a), and increases monotonously for P4VP(**1**)*_x_* as a function of azobenzene concentration. No significant differences can be observed in the inscription dynamics, and for P4VP(**1**)_1.0_ the diffraction efficiency reaches 34% within ca. 25 min of inscription. For P4VP(**2**)*_x_*, in turn, the inscription dynamics depends on the azobenzene concentration in a complicated manner (Figure 4b), first slowing down (*x* = 0.1 → *x* = 0.2) and eventually adapting a sigmoidal-type shape (*x* = 0.2 → *x* = 0.5 → *x* = 1.0), and reaching 35% diffraction efficiency for the equimolar complex upon ca. 1.5 h of irradiation. The faster SRG evolution of P4VP(**1**)*_x_* as compared to P4VP(**2**)*_x_* is further illustrated in Figure 4c, which presents the normalized diffraction efficiencies for the samples with *x* = 0.2 during the first 30 min of SRG inscription. Note that the saturation is reached faster even if the absorbance of P4VP(**1**)_0.2_ is lower than for P4VP(**2**)_0.2_. The lower absorbance indicates that the nominal complexation degree for the former may actually be lower than for the latter (some material may be lost, e.g., through the filtering process). Yet the result is in accordance with our earlier findings [20] and confirms that iodoethynylphenyl-capped azobenzenes outperform their tetrafluoroiodophenyl-capped counterpart in terms of the efficiency of SRG formation.

**Figure 5:**
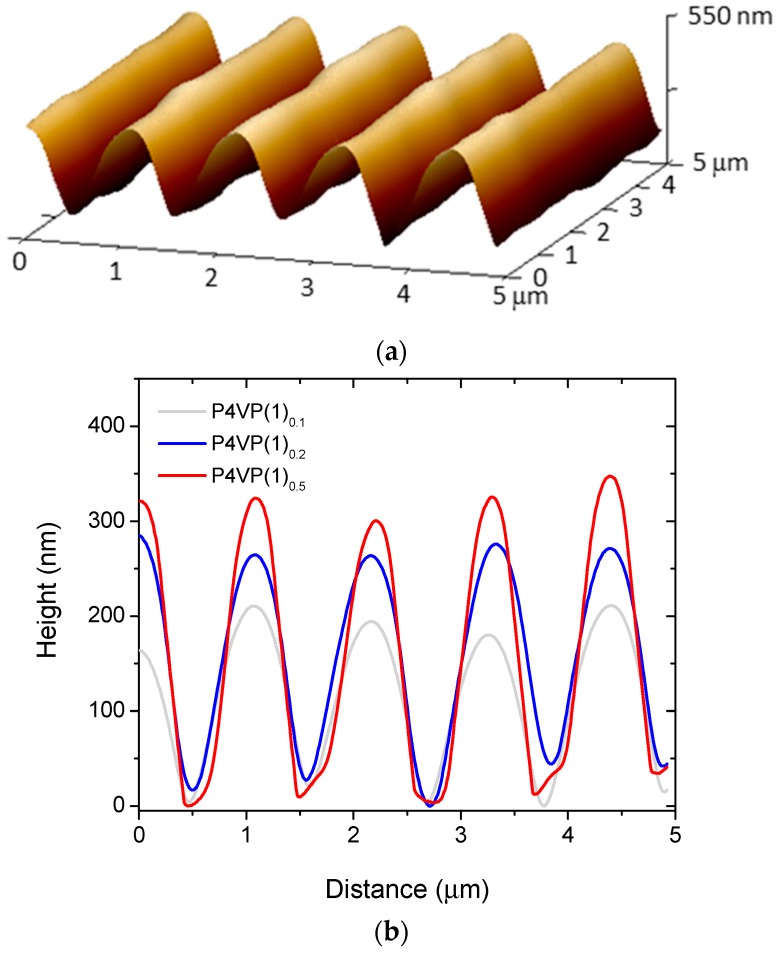
(**a**) 3D AFM image of P4VP(**2**)_0.1_ after 2 h of SRG inscription; (**b**) cross-section profiles of the SRGs on P4VP(**1**)_0.1_, P4VP(**1**)_0.2_, and P4VP(**1**)_0.5_.

The SRGs were further analyzed by means of atomic force microscopy (AFM), and 3D micrograph of an SRG inscribed onto P4VP(**2**)_0.1_ is given in Figure 5a, indicating high-quality SRG with uniform surface profile. We note that, in some cases, for instance P4VP(**1**)_0.5_ in Figure 5b, the surface profile at the through of the SRG was non-symmetric, which is probably an artifact due to the size of the measuring tip. Thereby, the actual modulation depths for some samples may even be higher as the values reported in Table 1. Figure 5b shows the SRG profiles for P4VP(**1**)_0.1_, P4VP(**1**)_0.2_, and P4VP(**1**)_0.5_, for which the modulation depth (alike the diffraction efficiency shown in Figure 4a) increases systematically with azobenzene concentration even if at the same time the sample thickness decreases (Table 1). For P4VP(**1**)_0.5_, the modulation depth even exceeds the initial sample thickness. For P4VP(**2**)*_x_*, for which the SRG inscription time was significantly longer, the modulation depths are generally higher than for P4VP(**1**)*_x_* but the correlation between the diffraction efficiencies and the modulation depths is less clear. This is especially the case for P4VP(**2**)_1.0_, yielding a 35% diffraction efficiency yet only a shallow, poorly defined SRG. This indicates that a significant contribution to the diffraction efficiency arises from refractive-index modulation in the bulk of the film, possibly as a result of light-induced modification of chromophore aggregates under high-intensity (300 mW·cm^−2^) irradiation [44]. The modulation depth obtained for P4VP(**2**)_0.5_, 390 nm, is the highest reported for halogen-bonded polymer–azobenzene complexes. Table 1 summarizes the properties of all the gratings studied in this work. The gratings are relatively stable over time, but after several months of storage, the DEs had slightly decreased. Samples with high chromophore concentration appeared more stable in time than the samples with lower complexation degree, yet this issue was not further elaborated.

## 3. Materials and Methods

Azobenzene **1** (*E*)-*N*,*N*-dimethyl-4-((2,3,5,6-tetrafluoro-4-iodophenyl)diazenyl)aniline and azobenzene **2** (*E*)-4-((4-(iodoethynyl)phenyl)diazenyl)-*N*,*N*-dimethylaniline were synthesized as described in the literature [13,14]. Poly(4-vinylpyridine) (P4VP) with a molecular weight of 1760 g/mol was purchased from Polymer Source, Inc. The compounds were separately dissolved in a mixture of chloroform and dimethylformamide (60:40 *v*/*v*) in a concentration of 4 wt % for the azobenzenes and 5 wt % for P4VP. The mother solutions were subsequently filtered, and mixed in proper amounts in order to yield the desired complexation degree.

The mixed solutions were spin-coated on clean microscope glass substrates at 1000 rpm for 60 s in a nitrogen atmosphere. Afterwards, the solvent was evaporated by heating the samples at 80 °C for 20 min. The optical micrographs were taken with a Canon EOS 70D camera mounted on a DM 4500P optical microscope (Leica Microsystems GmbH, Wetzlar, Germany). The UV-Vis absorption spectra were measured using a USB2000+ fiber-optic spectrometer and a DHL-2000-BAL light source (Ocean Optics, Largo, FL, USA). For easy comparison, the absorbances given in Figure 3 have been adjusted to match a thickness of 400 nm for each sample. The thickness of the films was determined using a Veeco Dektak 6 M stylus profilometer (Bruker, Billerica, MA, USA). Surface topographies were analyzed with a Veeco Dimension 5000 atomic force microscope with Nanoscope V controller (Bruker, Billerica, MA, USA). Silicon AFM tips (NSC 15/AIBS, MikroMasch, Tallinn, Estonia) coated with Al with a tip radius of 10 nm were used to probe the surface profiles.

The inscription of SRGs was performed using a custom-built Lloyd mirror interferometer setup and a Genesis CX488-2000 optically pumped semiconductor laser with a wavelength of 488 nm (Coherent Inc., Santa Clara, CA, USA). The laser beam was spatially filtered, expanded, and cut with an iris to a diameter of 8 mm. The intensity of the inscription beam was set to 300 mW·cm^−2^. The beam was linearly polarized, after which a quarter-wave plate was used to make the inscription beam circularly polarized. The circularly polarized, expanded beam was then directed to the interferometer, such that half of the beam impinged to the sample while the other half reflected on the sample surface from a mirror set at 90° angle to the sample plane, creating the interference pattern needed for the SRG inscription. In such a configuration, the polarization of the reflected half of the inscription beam is altered into an elliptical polarization [46]. Although in the resulting interference pattern both intensity and polarization change periodically, it is commonly applied, efficient in producing surface relief gratings, and produces comparable results between different samples. The fringe spacing of the interference pattern, Λ, and thereby the periodicity of the SRGs, is determined by the equation Λ = λ/(2sin*θ*), where λ is the wavelength of the writing beam and *θ* is the angle between the sample normal and the propagation axis of the writing beam. In this study, the periodicity was set to ca. 1 µm. All the SRGs were inscribed on freshly prepared samples in order to ensure a reliable comparison between the different samples without any potential effects brought about by sample aging.

## 4. Conclusions and Outlook

We have studied the SRG formation in halogen-bonded polymer–azobenzene complexes as a function of complexation degree. Halogen-bond-donating azobenzenes with tetrafluoroiodophenyl (**2**) and iodoethynylphenyl (**1**) groups have been complexed with poly(4-vinylpyridine), up to the equimolar complexation degree. At high azobenzene concentrations, **2** is more prone to phase separation as confirmed with optical microscopy and UV-Vis spectroscopy, presumably due to packing of fluorinated and non-fluorinated rings. The SRG inscription dynamics, followed by in-situ diffraction measurements, is systematically faster for **1**-based complexes than for **2**-based complexes, while the latter provides gratings with the largest modulation depth. Diffraction efficiencies exceeding 30% and modulation depths close to 400 nm are reported, which are much higher than those previously reported for halogen-bonded polymer–azobenzene complexes. All in all, this work demonstrates that SRGs with high modulation depth and high diffraction efficiency can be inscribed in halogen-bonded polymer–azobenzene complexes, at the same time pointing towards complex relation between chromophore aggregation, diffraction dynamics, and SRG evolution. Further studies on photoinduced anisotropy and SRG inscription using different light intensities are required to further clarify these issues and will be the topic of our future studies.

## Figures and Tables

**Table 1:** Summary of the SRG experiments, including the thicknesses of the films used, the diffraction efficiencies, and the modulation depths of the gratings (with an error margin of ±15 nm) as determined from the AFM images. The measurements were performed after completion of the SRG formation, which occurred between 1 and 2 h depending on the sample.

Sample	Film Thickness	Diffraction Efficiency	Modulation Depth
P4VP(**1**)_0.1_	380 nm	0.14	180 nm
P4VP(**1**)_0.2_	300 nm	0.23	245 nm
P4VP(**1**)_0.5_	263 nm	0.30	315 nm
P4VP(**1**)_1.0_	223 nm	0.34	315 nm
P4VP(**2**)_0.1_	397 nm	0.22	330 nm
P4VP(**2**)_0.2_	407 nm	0.28	360 nm
P4VP(**2**)_0.5_	278 nm	0.29	390 nm
P4VP(**2**)_1.0_	(330 nm) ^1^	0.35	(125 nm) ^1^

^1^ Values could not be measured in an accurate manner due to the phase separation.
